# 

**Published:** 1877-07

**Authors:** 


					﻿TZS r>\T3LjTF-TITL'D TN lF3f5E5.	___
VdLuih XV	JULY- (877-	' Nmujin 4.
i	ATLANTA
i	• I
Medical and Surgical Journal,
. (
i
______________ __	____ I
MONTHLY: THKEE DOLI.AKS HU: ANNl M. IN ADVANCE.
EriITOr< :
J. (4. WESTMOHELANI), M.D.
J'rofesfifir .Miiterhi Mcilieti hi Athnibi Meili.-nl Cnlleiie.
i ■	• ■■
j. .	________________
i	II. H. DICKSON, PimUSHI’ R.
PAX PT SCIENTIA, SKh 'vPIHTAS SIXE TIMOPE.
I
j	.	- ■*; -
1 • . ■ •
(	ATLANTA, (IKOh’GIA:
)	JI. 11. DicK.'iON, HOOK AM> .ton j-iny rm aki^ hookhindjir.
				

